# High power visible light emitting diodes as pulsed excitation sources for biomedical photoacoustics

**DOI:** 10.1364/BOE.7.001260

**Published:** 2016-03-14

**Authors:** Thomas J. Allen, Paul C. Beard

**Affiliations:** Department of Medical Physics and Biomedical Engineering, University College London, Gower Street, WC1E6BT, UK

**Keywords:** (110.5120) Photoacoustic imaging, (170.3880) Medical and biological imaging

## Abstract

The use of visible light emitting diodes (LEDs) as an alternative to Q-switched lasers conventionally used as photoacoustic excitation sources has been explored. In common with laser diodes, LEDs offer the advantages of compact size, low cost and high efficiency. However, laser diodes suitable for pulsed photoacoustic generation are typically available only at wavelengths greater than 750nm. By contrast, LEDs are readily available at visible wavelengths below 650nm where haemoglobin absorption is significantly higher, offering the prospect of increased SNR for superficial vascular imaging applications. To demonstrate feasibility, a range of low cost commercially available LEDs operating in the 420-620nm spectral range were used to generate photoacoustic signals in physiologically realistic vascular phantoms. Overdriving with 200ns pulses and operating at a low duty cycle enabled pulse energies up to 10µJ to be obtained with a 620nm LED. By operating at a high pulse repetition frequency (PRF) in order to rapidly signal average over many acquisitions, this pulse energy was sufficient to generate detectable signals in a blood filled tube immersed in an Intralipid suspension (µ_s_’ = 1mm^−1^) at a depth of 15mm using widefield illumination. In addition, a compact four-wavelength LED (460nm, 530nm, 590nm, 620nm) in conjunction with a coded excitation scheme was used to illustrate rapid multiwavelength signal acquisition for spectroscopic applications. This study demonstrates that LEDs could find application as inexpensive and compact multiwavelength photoacoustic excitation sources for imaging superficial vascular anatomy.

Published by The Optical Society under the terms of the Creative Commons Attribution 4.0 License. Further distribution of this work must maintain attribution to the author(s) and the published article’s title, journal citation, and DOI.

## 1. Introduction

Photoacoustic imaging is a hybrid imaging technique, which combines the high spectroscopic based contrast of optical imaging with the high spatial resolution (<100μm) of ultrasound imaging [1]. Q-switched Nd:YAG pumped OPO, Ti:Sapphire or dye laser systems are most commonly used as excitation sources in widefield photoacoustic tomography or acoustic resolution photoacoustic microscopy. These laser systems can provide the necessary mJ pulse energies with nanosecond pulse durations at biologically relevant wavelengths and have played an essential role in the development of photoacoustic imaging in the laboratory. However, their high cost, large physical size, cooling and maintenance requirements can limit their practical biomedical application.

**Fig. 1:**
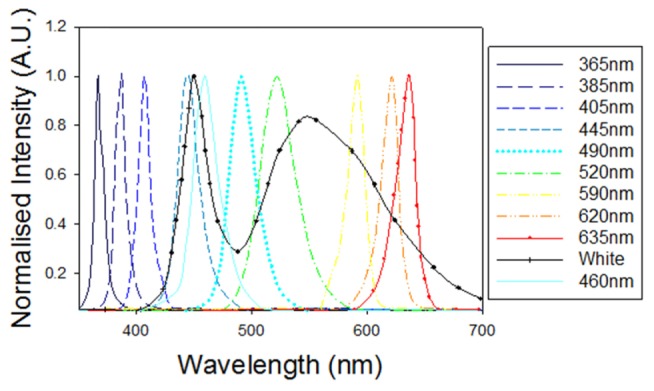
Optical spectra for a range of commercially available LEDs (Mightex Systems).

Laser diodes can address some of these drawbacks as they are relatively inexpensive, highly compact and power efficient. High peak power pulsed laser diodes (HPLD) of the type designed for LIDAR and other ranging applications have previously been used for photoacoustic imaging [2–4]. These studies suggest that, despite the much lower intrinsic pulse energy of a single element HPLD (~µJ) compared to Q-switched Nd:YAG based laser systems (mJ), it is possible to achieve adequate SNR for superficial imaging applications. This typically requires combining multiple emitters (e.g. stacking laser diodes can provide pulse energies in the mJ range [4]) and employing optimised signal processing strategies that exploit the ability to arbitrarily pulse modulate HPLDs at high repetition frequencies [2–4]. However, HPLDs are typically commercially available only at wavelengths greater than 750nm where haemoglobin absorption, the principal source of endogenous photoacoustic contrast, is low. LEDs could offer a useful alternative in this respect. They are available at shorter wavelengths below 650nm (see Fig. 1

) where haemoglobin absorption can be as much as an order of magnitude higher offering the prospect of significantly increased SNR for superficial vascular imaging. However, unlike HPLDs designed for LIDAR which can readily provide 100s of Watts over nanosecond timescales corresponding to tens of µJ pulse energies [2], most commercially available LEDs are specified for continuous wave operation at only a few Watts. In this study, we explore the prospect of overcoming this limitation by overdriving visible LEDs by a factor of 10 with short pulses and operating at a low duty cycle (<1%) [5]. We show that this strategy enables pulse energies of the order of 10µJ to be achieved over nanosecond timescales, more than a factor of 20 higher than previously reported [6] for an LED based photoacoustic source. These pulse energies are comparable to those of 905nm HPLDs used previously to obtain in vivo photoacoustic images [3], but with the advantage that haemoglobin absorption is significantly higher at the visible wavelengths of the LEDs used.

Section 2 demonstrates that photoacoustic signals can be generated and detected in a realistic tissue mimicking phantom when overdriving an LED and using widefield illumination. In Section 3, the LED is used in conjunction with a cylindrical scanner to illustrate its application to photoacoustic tomography. The spectroscopic capability of LEDs is demonstrated in Section 4 by using a four wavelength device to acquire single point measurements in a realistic tissue mimicking phantom. Section 5 illustrates how the use of coded excitation can be used to improve SNR and acquire photoacoustic signals at multiple wavelengths simultaneously.

## 2. Single point measurements in a tissue mimicking phantom

Figure 2(a)

**Fig. 2:**
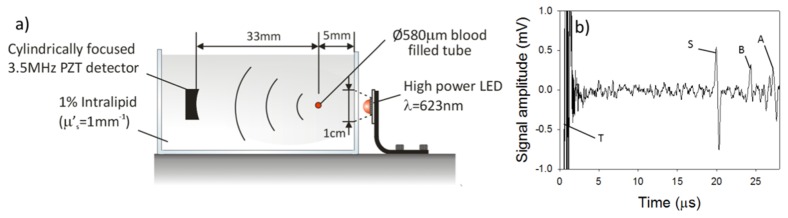
(a) Experimental setup, comprising a 580µm diameter blood filled tube immersed in intralipid (µ_s_’ = 1mm^−1^), (b) Photoacoustic signal (S) generated in a phantom. At t = 27μs a reflection (A) of the photoacoustic signal can be seen which is due the acoustic impedance mismatch between the intralipid and the wall of the tank. T denotes the trigger and B is a signal generated in the tank wall. P = 9µJ, N = 1000

shows the experimental setup used to investigate the use of an LED to generate and detect a photoacoustic signal in a realistic tissue mimicking phantom. The phantom comprised a 580µm diameter PMMA tube filled with human blood at a physiologically realistic concentration (35% haematocrit) and immersed to a depth of 5mm in a 1% suspension of intralipid, providing a reduced scattering coefficient of μ_s_’ = 1mm^−1^, which is comparable to that of soft tissues such as those in the breast [7] and skin [8]. The phantom was illuminated by a high power LED (SST-90 from Luminus), driven by a commercial driver (PCO-7120, Directed Energy, Inc.), which provided a pulse duration of 200ns, a peak current of 50A and a PRF of 500Hz. The duty cycle was 0.01%, which is well below the 1% duty cycle previously reported as safe (no noticeable damage to the device) when overdriving LEDs by 20 times their nominal current [9]. The optical pulse energy P and wavelength λ of the device were 9μJ and 623nm respectively, and the beam diameter incident on the intralipid solution was approximately 1cm. A cylindrically focused PZT detector (3.5MHz, V383 Panametric) with a focal length of 33mm was used to detect the photoacoustic signals and the blood-filled tube was placed at the focus of the ultrasound detector. The photoacoustic signal was amplified using a low noise voltage amplifier (60dB, Analog Modules Inc), averaged 1000 times and downloaded to a personal computer (PC). Figure 2(b) shows the detected photoacoustic signal for which the SNR was measured to be 7.

## 3. Photoacoustic imaging of a tissue mimicking phantom

Figure 3(a)

**Fig. 3:**
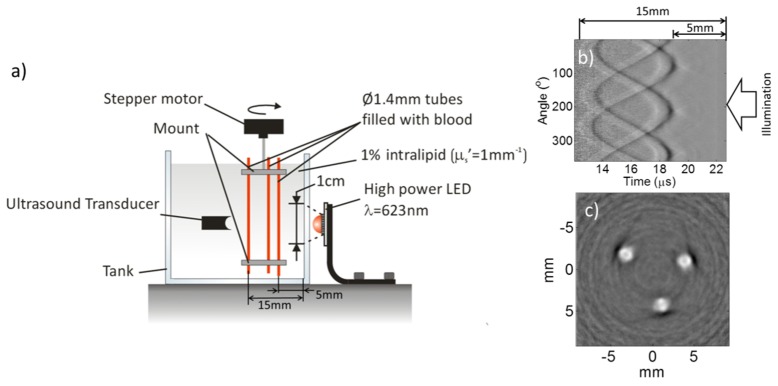
(a) Photoacoustic imaging setup (b) Time-resolved photoacoustic signals of three 1.4mm tubes filled with human blood (35% haematocrit) and immersed in 1% Intralipid (μ_s_’ = 1mm^−1^), (c) Reconstructed photoacoustic image. P = 9µJ, N = 5000.

shows a schematic of the experimental setup used to demonstrate the use of an LED for widefield photoacoustic tomography. The setup used the cylindrically focused PZT detector and LED described in Section 2. The LED driving parameters also remained the same. The phantom comprised three 1.4mm diameter PMMA tubes filled with human blood (35% haematocrit) which were immersed in 1% intralipid (μ_s_’ = 1mm^−1^). All three tubes were located in a mount which was rotated by 360 degrees in steps of 0.9 degrees using a stepper motor. The tubes were placed perpendicular to the focal plane of the ultrasound detector and within its focal zone. The LED was located on the opposite side of the ultrasound detector in order to operate in forward mode. The light emitted by the LED was transmitted through the transparent tank wall (Perspex) and formed an incident beam diameter of approximately 1cm on the intralipid suspension. The detected photoacoustic signals were averaged 5000 times. The time resolved photoacoustic signals were corrected for the exponential decay of light as a function of depth, and time reversal [10] was used to reconstruct a photoacoustic image.

Figure 3(b) shows the detected time resolved photoacoustic signals as a function of scan angle. It can be seen that photoacoustic signals corresponding to a penetration depth of up to 1.5cm are clearly visible. The photoacoustic image reconstructed from these signals is shown in Fig. 3(c) and clearly reveals all three tubes in the phantom. The tube diameters were measured from the reconstructed image and found to be within 0.1mm of their nominal values. The image contrast ratio was measured to be 11.5.

## 4. Multiwavelength measurements

**Fig. 4:**
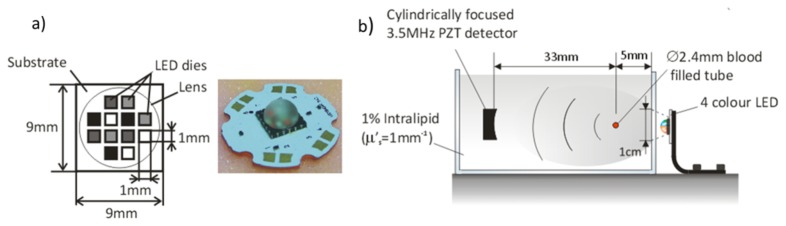
(a) Schematic and photograph of the four wavelength LED, (b) Experimental setup, comprising a 2.4mm diameter blood filled tube immersed in a solution of intralipid (µ’_s_ = 1mm^−1^).

Multiwavelength photoacoustic imaging has the ability to spectroscopically identify and quantify the concentration of specific chromophores [11, 12] such as oxyhemoglobin and deoxyhemoglobin. To demonstrate the potential spectroscopic capability of LED based devices, a compact (total foot print: 12mm^2^) four wavelength device (LZC-03MA07, from LedEngin) was used to obtain photoacoustic spectra in a tissue mimicking phantom. The LED comprised 4 groups of 3 LEDs (see Fig. 4(a)

), each set emitting at one of the following wavelengths: 460, 530, 590 and 620nm with respective pulse energies of 1.8, 0.4, 1.7, 2.7μJ. To achieve these pulse energies the LEDs were overdriven by ten times their CW current limit. The pulse duration and PRF were 200ns and 500Hz respectively.

Figure 4(b) shows the experimental setup. A 2.4mm diameter tube was filled with human blood (35% haematocrit) and immersed to a depth of 5mm in a 1% suspension of intralipid (µ_s_’ = 1mm^−1^). The multi-wavelength LED illuminated one side of the tube, and a cylindrically focused PZT detector (3.5MHz, V383 Panametric) of focal length 33mm was placed on the opposite side to detect the generated photoacoustic signals. The detected signals were amplified using a preamp (8dB, Precision Acoustics Ltd) and a low noise voltage amplifier (40dB gain, model 322-8-B-50 Analog module Inc) and averaged 10000 times.

Figure 5

**Fig. 5:**
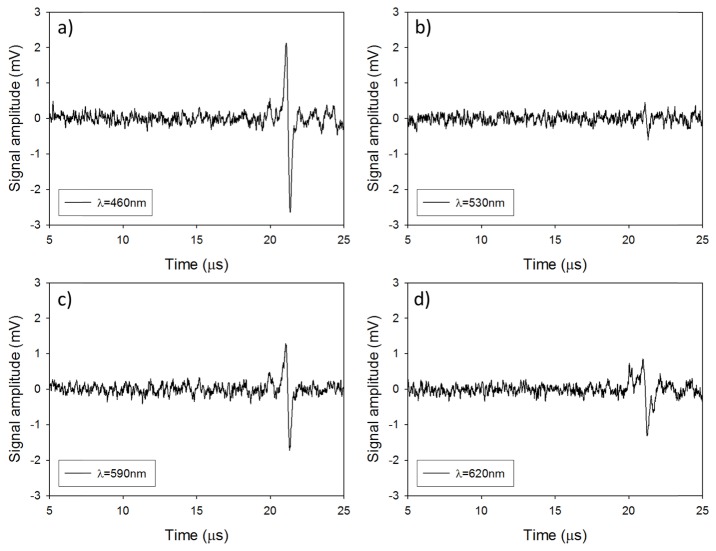
Photoacoustic signals generated in a realistic tissue mimicking phantom at (a) λ = 460nm, P = 1.8µJ, N = 10,000 (b) λ = 530nm, P = 0.4 µJ, N = 10,000 (c) λ = 590nm, P = 1.7 µJ, N = 10,000 and (d) λ = 620nm, P = 2.7 µJ, N = 10,000.

shows the generated photoacoustic signals for each of the four wavelengths. The SNR was measured to be 5.7, 1.59, 4.15, and 3.5 at 460, 530, 590, and 620nm respectively.

**Fig. 6:**
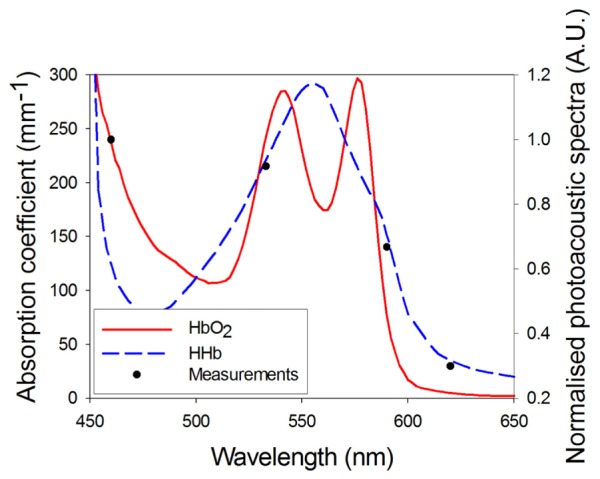
Absorption spectra of oxy and deoxy haemoglobin overlaid by the photoacoustic spectra obtained from the blood filled tube using the four wavelength device.

The peak amplitudes of the detected photoacoustic signals were normalised to their respective pulse energy and plotted as a function of wavelength as shown in Fig. 6

alongside the absorption spectra of oxy- and deoxy-haemoglobin. Although the oxygenation level of the blood filled tube was unknown, the relative trend in the measured photoacoustic spectra is broadly consistent with the absorption spectra of oxy- and deoxy-haemoglobin.

As previously mentioned, the main challenge in using LEDs is to overcome the low SNR of the generated photoacoustic signals. Using a multi-wavelength device, as opposed to a single wavelength device, can further limit the pulse energy, as the total emitting area of the device will be shared between wavelengths. For example, the single wavelength SST-90 device used in Sections 2 and 3 had a 9mm^2^ emitting area, whereas the multi-wavelength device used in this section had a total emitting area of 12mm^2^ divided into four individual emitting elements (one for each wavelength), each corresponding to a 3mm^2^ area. Since the pulse energy scales with emitting area, the pulse energy provided at a single wavelength by the multi-wavelength device will be 3 times lower than that provided by a larger area single-wavelength device. However there is the potential to mitigate this by driving all four elements simultaneously [13] and using the frequency spectrum of the resulting photoacoustic signal to design a Wiener filter. The filter can then be applied to the signals generated using a single element in order to supress those frequency components associated with the noise, and retain only those that contribute to the signal thereby improving SNR. Implicit in this approach is the assumption that the bandwidth of the Wiener filter is sufficiently wide to accommodate the full extent of the different frequency spectra of the signals generated at each wavelength. This appears intuitively reasonable given that the filter design is derived from the photoacoustic signal generated at all four wavelengths simultaneously.

**Fig. 7:**
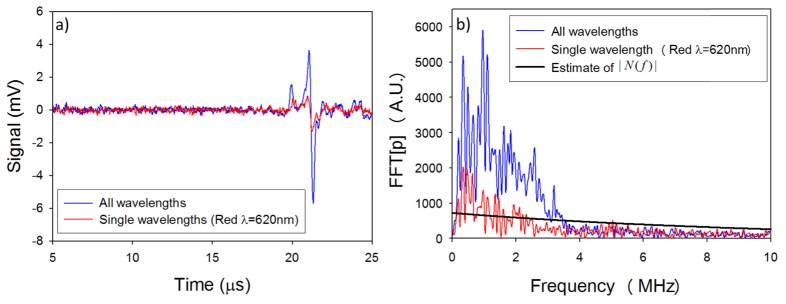
Photoacoustic signals obtained when driving all four wavelength simultaneously and for a single wavelength at λ = 620nm. a) Time domain signals b) frequency domain signals.

To illustrate the use of a Wiener filter in this way, all four elements were driven simultaneously and the time domain photoacoustic signal and its corresponding frequency spectrum are shown in Fig. 7(a)

(blue curve). For comparison the photoacoustic signal obtained using the 620nm emitting element alone (red curve) is also shown in Fig. 7(a). The power spectrum ${\left|C\left(f\right)\right|}^{2}$of the detected photoacoustic signal generated by all four wavelengths can be expressed as:

$${\left|C\left(f\right)\right|}^{2}={\left|S\left(f\right)\right|}^{2}+{\left|N\left(f\right)\right|}^{2}$$ (1)

where ${\left|S\left(f\right)\right|}^{2}$ is the power spectrum of the noise free photoacoustic signal generated by all 4 wavelengths and ${\left|N\left(f\right)\right|}^{2}$ is the noise power spectrum. A Wiener filter H(f) is defined as:

$$H\left(f\right)=\frac{{\left|S\left(f\right)\right|}^{2}}{{\left|S\left(f\right)\right|}^{2}+{\left|N\left(f\right)\right|}^{2}}$$ (2)

and can then be used to filter a photoacoustic signals generated by a single wavelength. This filter approaches zero when ${\left|N\left(f\right)\right|}^{2}>>{\left|S\left(f\right)\right|}^{2}$, and one when ${\left|N\left(f\right)\right|}^{2}<<{\left|S\left(f\right)\right|}^{2}$. Using Eq. (1) and 2, H(f) can be rewritten as a function of the power spectrum of the measured photoacoustic signal${\left|C\left(f\right)\right|}^{2}$ and the noise ${\left|N\left(f\right)\right|}^{2}$:

$$H\left(f\right)=\frac{{\left|C\left(f\right)\right|}^{2}-{\left|N\left(f\right)\right|}^{2}}{{\left|C\left(f\right)\right|}^{2}}$$ (3)

To design the filter H(f), an estimate of ${\left|N\left(f\right)\right|}^{2}$ is required. This can be obtained by identifying a part of the time domain signal not containing any photoacoustic signal (for example, the part before the trigger), calculating the Fourier transform, and fitting a curve to the peaks of the Fourier spectrum. The resulting estimate of |N(f)| is plotted in Fig. 7(b).

**Fig. 8:**
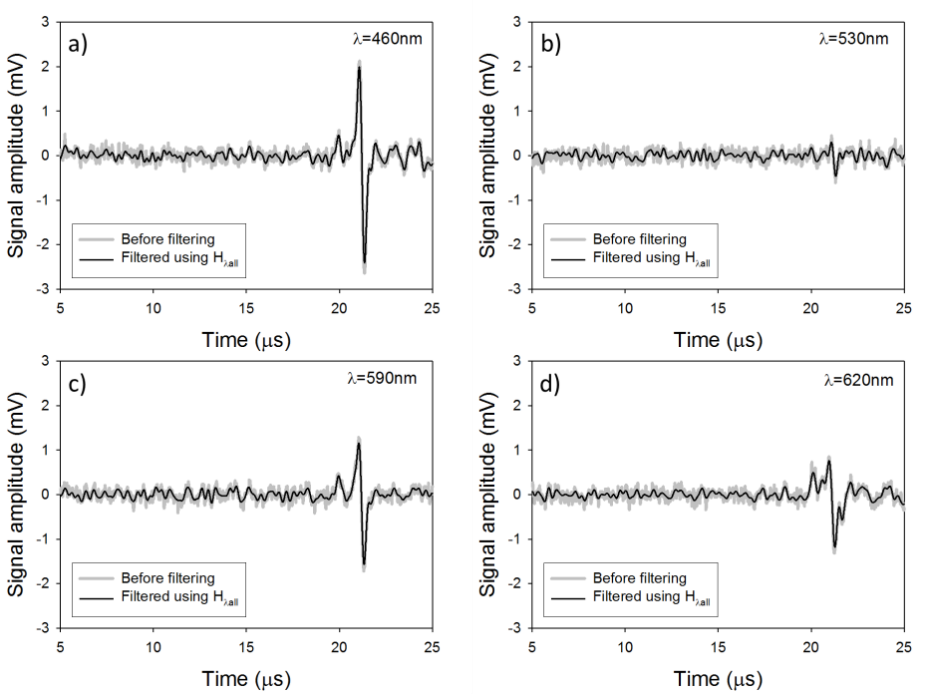
Photoacoustic signals generated in a realistic tissue mimicking phantom before and after filtering at (a) λ = 460nm, (b) λ = 530nm, (c) λ = 590nm and (d) λ = 620nm.

Filtering the photoacoustic signals generated by a single wavelength ($C_{\lambda_{1}}$) then yields:

$$Y_{\lambda_{1}}(t)=IFFT[FFT{\left(C_{\lambda_{1}}(t)\right)}_{}\times H(f)]$$ (4)

where $Y_{\lambda_{1}}$denotes the filtered signal. Such a filter will strongly attenuate the frequency components which do not contribute to the signals, and preserve those which do. Figure 8

shows the photoacoustic signals before and after being filtered using H(f). Before filtering, the SNRs of the photoacoustic signals were 5.7, 1.59, 4.15 and 3.5 at 460nm, 530nm, 590nm and 620nm, respectively. After filtering, the SNRs were 11.8, 2.2, 6.8 and 6, thus corresponding to an average SNR improvement of a factor of 1.7 compared to that obtained in Fig. 5.

## 5. Coded excitation

The potential to arbitrarily modulate LEDs is a significant advantage compared to conventional Q-switched photoacoustic excitation laser sources, as it allows a variety of coded excitation schemes to be implemented. Such schemes provide the opportunity to increase the SNR of the photoacoustic signal as a function of $\sqrt{N}$,where N is the number of bits within the code. Coded excitation schemes can also be used to acquire photoacoustic signals at multiple wavelengths simultaneously by using a different orthogonal code for each wavelength, thus reducing acquisition time [14, 15].

**Fig. 9:**
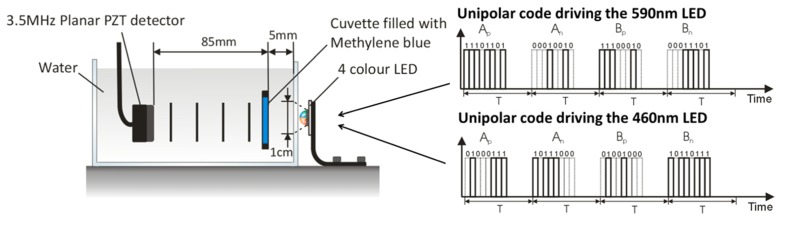
Experimental setup and an example of a Golay excitation code (N = 8bit) based on transmission of four unipolar codes: A_p_/A_n_ and B_p_/B_n_ represent the positive (1) and negative (−1) bits of the biphase code A and B respectively. T is the time delay between consecutive unipolar codes.

To illustrate the implementation of coded excitation schemes using LEDs, photoacoustic signals were generated using Golay codes similar to those used to drive laser diode based photoacoustic excitation source [15, 16]. The scheme required that two biphase codes (e.g. A and B) of N bits, which can be positive or negative, are transmitted [16, 17]. To generate biphase codes, two unipolar codes representing the positive (e.g. A_p_ for code A and B_p_ for code B, see Fig. 9(a)

) and negative (e.g. A_n_ for code A and B_n_ for code B) bits are transmitted. The generated photoacoustic signals (P_Ap_ and P_An_ for code A and P_Bp_ and P_Bn_ for code B) are then subtracted (e.g. P_Ap_-P_An_ and P_Bp_-P_Bn_). The biphase codes are then cross-correlated with their respective photoacoustic signals and summed ((A_p_-A_n_)★(P_Ap_-P_An_) + (B_p_-B_n_)★(P_Bp_-P_Bn_)) in order to recover the desired signal. It is required that a total of four unipolar codes (A_p_, A_n_, B_p_, B_n_) are transmitted to obtain a complete data set (see Fig. 9).

The experimental setup shown in Fig. 9 was used to generate photoacoustic signals; the four colour LED described in the previous section was used. The setup comprised a cuvette filled with methylene blue (µ_a_ = 1.1 and 16mm^−1^ at λ = 460 and 590nm, respectively) immersed in water to a depth of 5mm. The generated photoacoustic signals were detected using a planar PZT detector (3.5MHz, V381 Panametrics). Signals were amplified using a low noise voltage amplifier (Femto, 40dB) and downloaded to a PC. In this experiment, the LED elements were overdriven by only three times their rated current to reduce the likelihood of damage due to the increased duty cycle. Two different wavelengths (460nm and 590nm) of the four colour LED were driven with two different sets of orthogonal Golay codes simultaneously (shown in Fig. 9). Each Golay code was composed of four unipolar codes (A_n_, A_p_, B_n_ and B_p_) of N = 8bits, and the time delay between consecutive unipolar codes was set to T = 5ms. The record length of the arbitrary wave function of the signal generator used was limited to 5000 points, for a time interval of 5ms this corresponds to a sampling interval of 1μs and therefore each bit was 1µs long.

**Fig. 10:**
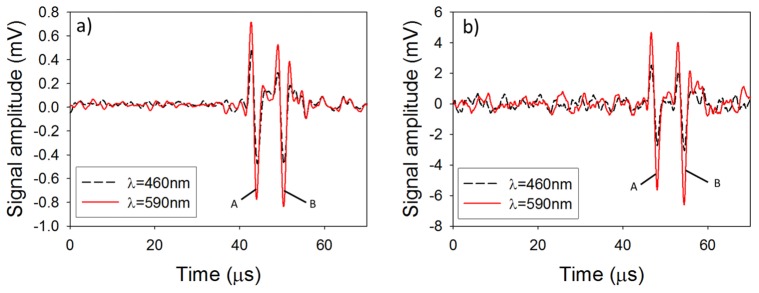
Photoacoustic signal generated via (a) Golay codes (b) pulsed excitation. A and B denote the photoacoustic signal and an acoustic reflection generated at the water air interface, respectively.

The photoacoustic signals obtained using the Golay excitation method when emitting at both wavelengths simultaneously are shown in Fig. 10(a)

. The photoacoustic signals obtained using the conventional pulsed excitation method when emitting sequentially at each wavelength are shown in Fig. 10(b). The temporal shape of the photoacoustic signals obtained with Golay excitation agree well with those obtained using the pulsed excitation. The SNR of the photoacoustic signals generated using Golay codes was 59 and 95 for 460nm and 590nm, respectively compared to 18 and 31 when using the pulse excitation method. This increase in SNR is consistent with an improvement of $\sqrt{N}$, where N is the number of bits within the code (in this example N = 8). The total acquisition time, when using the Golay excitation method, was 20ms (4 unipolar codes each lasting 5ms), and 10ms (2 excitation pulses, one for each wavelength, with a time delay between pulses of 5ms) when using the conventional pulsed excitation method. However, to achieve the same SNR using the conventional pulsed excitation method, the detected photoacoustic signals would need to be signal averaged N times resulting in a total acquisition time of 80ms.

## 6. Discussion and conclusion

This study has explored the use of visible LEDs as photoacoustic excitation sources. By over-driving with short high current pulses and operating at a low duty cycle, it has been demonstrated that pulse energies approaching 10µJ can be achieved using low cost (<$50) commercially available visible LEDs. By employing a high PRF in order to rapidly signal average, this enabled blood filled tubes immersed to a depth of 15mm in Intralipid to be detected. This suggests that *in vivo* imaging of the vasculature to depths of a few mm should be possible using a visible LED based photoacoustic excitation source. Note that large illumination beam diameters (>1cm) were used in the current study to illustrate the potential for use in widefield photoacoustic tomography. A previous study used a visible LED emitting at 623nm as an excitation source for single point measurements. However, its low pulse energy (400nJ) meant that focusing was required to generate a detectable photoacoustic signal thus limiting its practical application [6].

A novel highly compact four-wavelength LED was also evaluated to illustrate the potential for making multiwavelength photoacoustic measurements for spectroscopic applications. To mitigate the reduced pulse energy at each wavelength, two excitation strategies were investigated. The first used the signal generated using all four wavelengths simultaneously to design a Wiener filter which was then used to filter the signals generated at each individual wavelength to increase SNR. The second method used extended Golay coded excitation to increase SNR. In addition, by using different sets of orthogonal codes to drive elements emitting at different wavelengths, the simultaneous acquisition of photoacoustic signals at two wavelengths was demonstrated. This illustrates the potential of coded excitation schemes to provide faster acquisition than conventional sequential acquisition of photoacoustic signals at multiple wavelengths.

Even with future advances in signal processing and the realisation of Moore’s law type power scaling projections, it will be challenging for LED sources to cost-effectively match the output of conventional Q-switched Nd:YAG based excitation lasers. However this study suggests LEDs could find a role in superficial vascular imaging where the pulse energy requirements are relatively modest. In this context, a compact hand-held integrated LED based device employing widefield photoacoustic tomography for non-invasive dermatological investigations or oximetry type measurements can be envisaged. Applications may also lie in photoacoustic endoscopy or acoustic resolution microscopy [18] where the excitation beam is confined to some extent thereby further reducing the pulse energy requirements. LEDs may also be suitable excitation sources for OR-PAM; the pulse energies achieved in this study are certainly sufficient although the large emitting area of the LEDs used would make it challenging to achieve the necessary micron scale diffraction limited spot sizes. In all of these applications, visible LEDs could potentially be used in conjunction with near infrared laser diode sources to access a wide range of biologically relevant wavelengths in the 600-900nm spectral range for spectroscopic based discrimination or quantification (eg blood oxygenation). In summary it is considered that this work represents a step towards the development of compact, inexpensive visible and near infrared multiwavelength excitation sources for photoacoustic imaging.
